# Perks of a Structured Rehabilitation in Suprascapular Neuropathy Injury Post Proximal Humerus Fracture: A Case Report

**DOI:** 10.7759/cureus.60509

**Published:** 2024-05-17

**Authors:** Ritika S Bhagwani, Snehal Samal, Prishita Koul, Samruddhi M Karanjkar, Roshni R Nandanwar

**Affiliations:** 1 Department of Neuro Physiotherapy, Ravi Nair Physiotherapy College, Datta Meghe Institute of Higher Education and Research, Wardha, IND

**Keywords:** physiotherapy, case report, nerve injury, humerus, fracture, rehabilitation, neuropathy

## Abstract

Road traffic accidents lead to extensive damage to superficial as well as deep components in the body. Neurological structures that are affected due to open injuries have major impairments in the day-to-day life of an individual. High trauma incidents lead to nerve injuries, which are a common occurrence secondary to fractures after such falls. Nerve entrapment, nerve compression, nerve denervation, or demyelination usually result in the wasting of muscles supplied by it, which eventually causes muscle atrophy. Muscle atrophy limits the ability of an individual to move the extremities to achieve functional activities. Sensory neuropathy, in addition to motor neuropathy, is an associated complication. Physical therapy interventions are observed to play a significant role in nerve and muscle injury rehabilitation courses, thus improving quality of life. This report presents a case of a 43-year-old male who came to the hospital with complaints of pain and inability to move the shoulder after his bike was hit by a truck from behind and he experienced a fall. The patient presented to an orthopedic surgeon who took X-ray, electromyography (EMG), and nerve conduction velocity (NCV) investigations and confirmed fracture of greater tuberosity of the humerus and motor neuropathy of the suprascapular nerve. He was surgically treated and was referred to the physiotherapy outpatient department for postoperative management. A well-planned physical therapy program aimed to improve the range of motion and strength of the affected shoulder joint while preventing atrophy, thus improving quality of life.

## Introduction

Proximal humerus fractures are a common occurrence and a predicament for any individual. They rank as the seventh most common type of fractures in people. According to several studies carried out in various groups, their prevalence ranges from 4% to 10% of all fractures. High-energy trauma was present in only 3.6% of cases (23 patients, including 13 women and 10 males), with low-energy trauma making up the majority (96.4%) of the cases. Age was found, grouped by five-year intervals, to have a statistically significant association with the type of trauma (p = 0.001). Patients under 60 years were the majority for high-energy injuries (73.9%), while this age group only saw 20.7% of low-energy injuries [1]. However, across all injury mechanisms, men were more susceptible to high-energy fractures than females. Although open fractures of the proximal humerus are uncommon, displaced fractures can result in swelling, significant ecchymosis, tenting of the epidermis, or pressure necrosis [2]. Neurologic or vascular injuries are most common in cases of fracture dislocations. Displaced fracture of greater tuberosity alters the biomechanics, eventually damaging the rich network of nerve supply via the brachial plexus, hence manifesting neurological injury at humeral-scapular musculature. Nerve injury can cause paresis and inability to move the arm [3]. It is a major factor influencing recovery. Similar to the shoulder injury, the process of the associated nerve damage causes traction lesions of the nerves stretched over the head of the humerus during the head dislocation. The suprascapular nerve has a brief distance between its origin at the supraclavicular plexus and its anchorage point at the suprascapular notch, which can be damaged by traction. Abduction that goes beyond the physiological limit of mobility results in lesions of this nerve. A suprascapular nerve injury manifests as a generalized shoulder discomfort and a corresponding weakness in shoulder external rotation and abduction. Eventually, the suprascapular nerve-supplied shoulder girdle muscles may atrophy [4]. Indirect mechanical compression from an underlying anatomic variation, a space-occupying lesion, or repetitive traction and irritation of the nerve at one of the anatomically predisposed sites of suprascapular nerve focal entrapment, as described below, are thought to be the primary causes of muscle wasting, atrophy, and degeneration [5]. The suprascapular nerve provides a rich supply to two of the five rotator cuff muscles, i.e., supraspinatus and infraspinatus. Damage or injury to this nerve leads to complete or partial denervation of the musculature, which eventually results in atrophy or wasting. Combined injury to other nerves of the brachial plexus network due to proximal humerus fracture draws complications like rotator cuff muscle atrophy.

The present study describes the case of a 43-year-old man who suffered a shoulder injury through a motorcycle accident and experienced pain restricting movement and bruising. He visited an osteopath and applied some ointment for two weeks. Since no relief was obtained, he visited the hospital, and X-rays, electromyography (EMG), nerve conduction velocity (NCV), and MRI were done, which revealed a fracture of the greater tuberosity and motor neuropathy of the suprascapular nerve. He underwent surgical management and was referred to the physiotherapy outpatient department (OPD). An integrated physiotherapy plan was aimed at restoring the range of motion and strength of muscles.

## Case presentation

Patient information

A 43-year-old man came to a tertiary care rural hospital with complaints of pain and inability to move his left shoulder for two weeks. The patient reported a history of a road traffic accident on January 18, 2023, when he was driving a two-wheeler and was hit by a car from behind, due to which he lost his balance and fell and ended up injuring his left shoulder. He immediately developed pain and swelling over his shoulder and got aggravated by any attempts to make movements. The patient was taken to an osteopath by his friend, who applied some ointment, and after a few days of removing it, he noticed bruising and redness, which worried him. He then came to the orthopedic surgeon on February 2, 2023, who took the X-rays and suggested a displaced fracture of greater tuberosity of the left shoulder. An EMG and NCV were performed, which revealed motor neuropathy of the suprascapular nerve. EMG report confirmed partial denervation of the deltoid, supraspinatus, biceps, triceps, and rhomboids. He underwent open reduction and internal fixation, the affected shoulder was immobilized in a cast, and later, he was referred to physiotherapy OPD for post-surgical management.

Clinical examination

An informed consent was taken from the patient prior to the examination. On inspection, the patient was found in a sitting position and was oriented to time, place, and person. Bruising and redness were observed over the left shoulder. The swelling was also present. The affected upper extremity’s elbow joint was flexed across the chest and supported by the unaffected upper extremity. On palpation, grade 3 tenderness was found over the anterior joint line of the left shoulder. The temperature of the affected shoulder was raised slightly compared to the unaffected shoulder. All the findings below were recorded postoperatively after the removal of the cast. The range of motion is mentioned in Table 1. In Table 2, manual muscle testing findings of the patient are depicted, and findings of deep tendon reflexes are mentioned in Table 3. In Table 4, the sensory examination of the patient is presented. A special test, i.e., the empty can test, was performed in which the patient experienced pain while moving his arm against resistance and the contraction was weak.

**Table 1:** Range of motion examined bilaterally.

Movements	Right side	Left side
Shoulder		
Flexion	0˚-170˚	0˚-70˚
Extension	0-50	0-30
Abduction	0˚-175˚	0˚-40˚
Horizontal adduction	0˚-60˚	0˚-40˚
Medial rotation	0˚-75˚	0˚-15˚
Lateral rotation	0˚-80˚	0˚-10˚
Elbow joint		
Flexion	0˚-145˚	0˚-100˚
Extension	145˚-0˚	100˚-0˚

**Table 2:** Manual muscle testing findings bilaterally.

Movements	Right	Left
Shoulder movements		
Flexion	4/5	3-/5
Extension	4/5	4-/5
Abduction	4/5	3-/5
Adduction	4/5	3-/5
Medial rotation	4/5	3-/5
Lateral rotation	4/5	3-/5
Elbow movements		
Flexion	4/5	3-/5
Extension	5/5	3-/5

**Table 3:** Reflexes examination findings bilaterally.

Reflexes	Right	Left
Biceps jerk	2+	1+
Triceps jerk	2+	1+

**Table 4:** Sensory examination findings bilaterally.

Sensations	Right	Left
Touch	+2	+2
Temperature	+2	+2
Pain	+2	+2

Radiological findings

**Figure 1:**
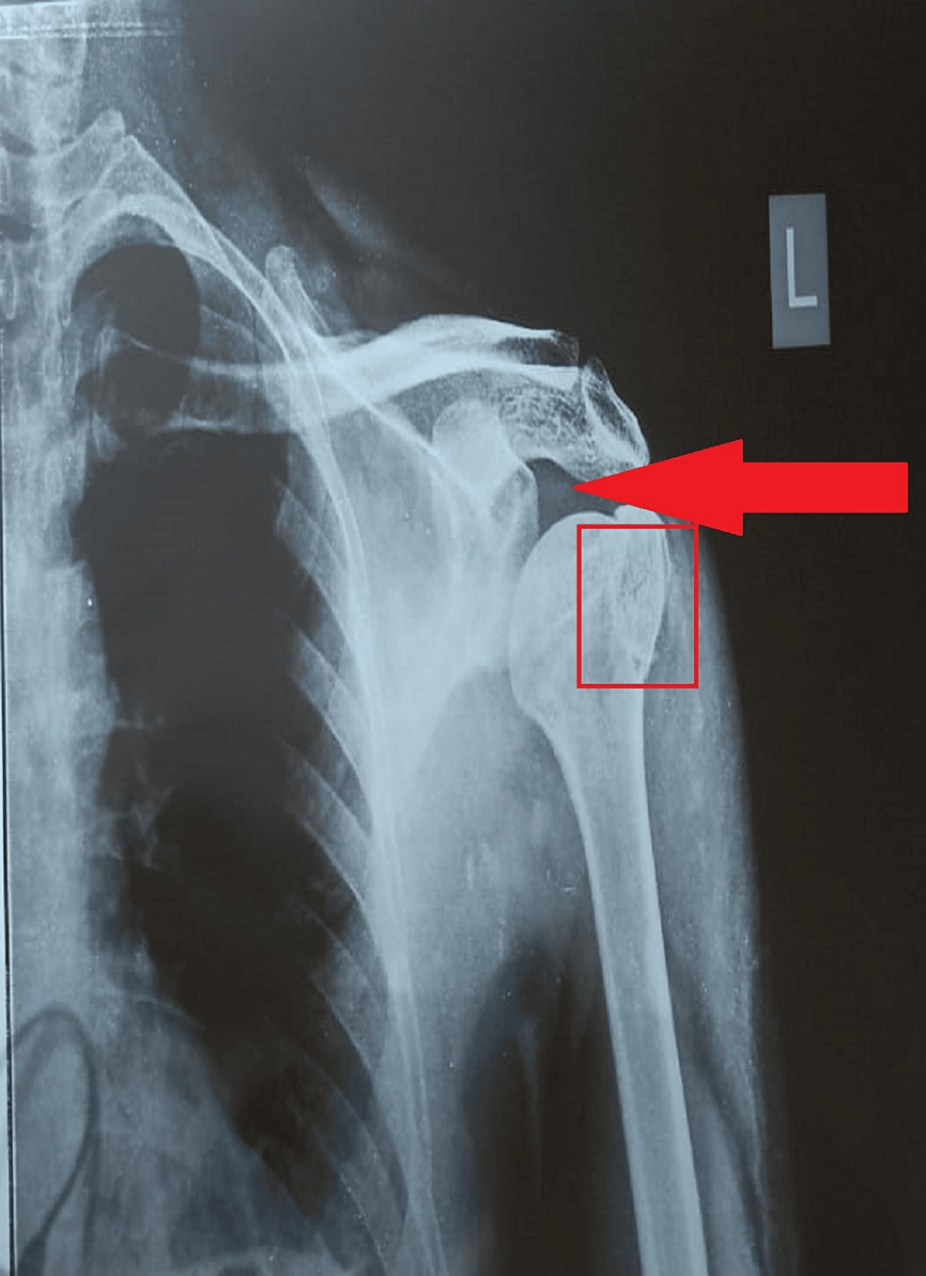
Preoperative anteroposterior X-ray of the left shoulder joint presenting a fracture of greater tuberosity of the humerus with slight displacement. The arrow depicts the displacement of the humeral head and the box depicts the fracture of greater tuberosity.

**Figure 2:**
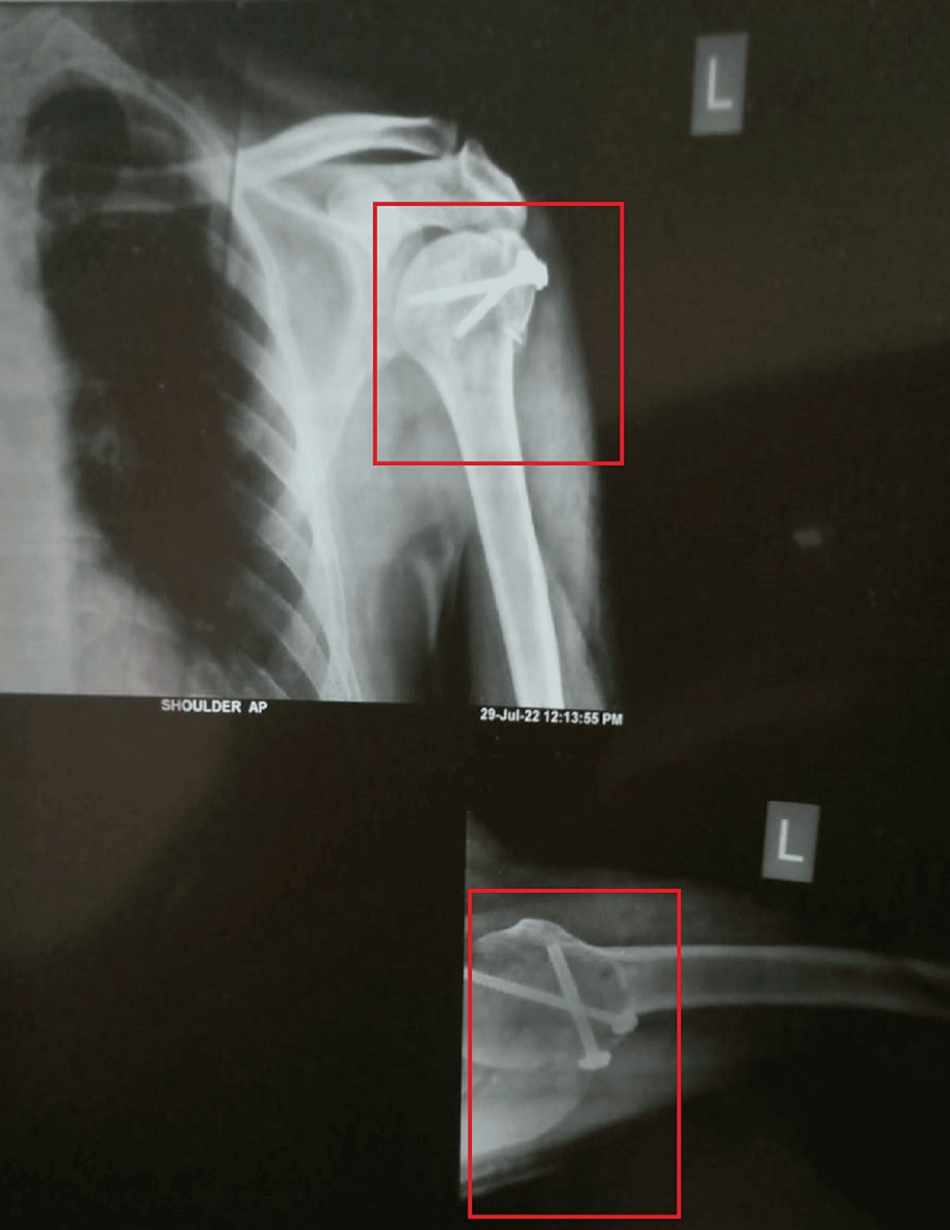
Postoperative anteroposterior X-ray of the left shoulder joint presenting internal fixation with screws for fracture of greater tuberosity of the humerus, reducing the displacement.

Figures 1, 2 show the findings of the X-ray of the left shoulder joint.

Physiotherapy management

The patient was referred to neuro physiotherapy OPD after the removal of his cast. He was treated in his post-immobilization phase. The physiotherapy intervention is mentioned in Table 5.

**Table 5:** Physiotherapy management over the course of two months. EMS: electrical stimulation; PNF: proprioceptive neuromuscular facilitation; reps: repetitions; D1/2: diagonal pattern.

S. No.	Goals	Physiotherapy interventions	Repetitions
1.	Patient education	Educating the patient about his impairments and the significance of physiotherapeutic management.	Once a week
2.	Initiate voluntary contraction of shoulder muscles	EMS-surged faradic current stimulation, giving 30 contractions to motor points of the deltoid, rhomboids, biceps, and triceps. PNF pattern D1 and D2 flexion, extension beginning with rhythmic initiation.	30 contractions for each muscle, 3 sets a day. 10 repetitions × 3 sets, twice daily
3.	Nerve conservation	Stimulation of suprascapular nerve along its course.	3 sets each day
4.	Reduce post-immobilization stiffness	Rigorous capsulolabral stretching. Vigorous passive range of motion for the shoulder joint.	5 reps × 1 set, 7 days a week; 10 reps × 1 set, 7 days a week
5.	Improving scapular strength	Scapular strengthening exercises such as rowing exercises, prone lying exercises of the shoulder at the edge of the bed, sitting ball press, etc. Scapular closed kinematic chain exercises like wall push-ups progressing to ground push-ups, scapular clocks, low row exercises, theraband scapular adduction, and shoulder yellow theraband exercises progressing to increased resistance of green theraband. Isometric exercises to deltopectoral musculature.	10 repetitions × 1 set, thrice daily. Initiated with 5 reps × 2 sets, progressing to 10 reps × 2 sets
6.	Strengthening of elbow joint musculature	Biceps and triceps muscle training initiated with theraband for elbow extension by stretching theraband by attaching overhead to the wall and flexion of the elbow by stretching theraband from underneath the feet. Using dumbbells to progress strengthening. Using wrist exerciser.	10 reps × 1 set, twice daily. 10 reps × 1 set, daily

Outcome measures

The following outcome measures were documented, as mentioned in Table 6.

**Table 6:** Outcome measures recorded pre and post-rehabilitation. SPADI: Shoulder Pain and Disability Index; DASH: Disabilities of the Arm, Shoulder, and Hand.

S. No.	Outcome measures	Pre-rehab	Post-rehab
1.	SPADI scale	55/100	40/100
2.	Range of motion	Shoulder flexion: 0˚-70˚; shoulder abduction: 0˚-40˚; external rotation: 0˚-10˚; medial rotation: 0˚-15˚; elbow flexion: 0˚-100˚	Shoulder flexion: 0˚-90˚; shoulder abduction: 0˚-55˚; external rotation: 0˚-20˚; medial rotation: 0˚-25˚; elbow flexion: 0˚-110˚
3.	Manual muscle testing	Shoulder: 3-/5; elbow: 3-/5	Shoulder: 4-/5; elbow: 4-/5
4.	DASH	45/100	35/100

## Discussion

Road traffic accidents are violent incidents that bring a lot of damage to the body and impair its functioning. To reduce the repercussions, physiotherapy assessment and management play a vital role. Salvini et al., in their review, highlight the significant outcomes that were obtained upon application of EMS and passive stretching to prevent muscle atrophy and restore muscle strength [3]. Solomen et al., in their case report, concluded that the application of various physiotherapy approaches, including muscle stimulators, progressive resisted exercises, sensory re-education, etc., showed signs of full recovery in six months [6]. As described in the current study, a 43-year-old man had an accident and came to the hospital with complaints of pain and restricted movement after two weeks. He was diagnosed with a fracture of greater tuberosity of the humerus and EMG and NVC investigations suggested demyelinating motor neuropathy of the suprascapular nerve, after which he was surgically operated on and referred to the physiotherapy department for postoperative management. de Santana Chagas et al. in their review discussed the significance of manual therapy, strength training, electrotherapy, and kinesiotherapy techniques application post brachial plexus injury [7]. On the basis of evidence-based studies, the patient in the current case was managed by similar categories of treatment methods and progression was recorded over the course of two months. Chagas et al. conclude the effectiveness of proprioceptive neuromuscular facilitation (PNF) when applied in the rehabilitation plan of injury to the upper brachial plexus and highlight physical therapy as a fundamental role player in functional recovery [8,9]. After 60 days of the rehabilitation program, the patient was discharged and continued physiotherapy in a local clinic. The patient was assessed post physical therapy management and positive outcomes were documented. As mentioned in the outcome measures, the patient reported improvement in doing activities of daily living, hence quality of life was improved.

## Conclusions

Post-fracture complications, such as neuropathy, are critical as they hamper activities of daily living. The above case describes the disability of the patient who was experiencing postoperative humerus fracture, which is why he was referred to physiotherapy OPD. The positive effects of physiotherapy interventions in the above case were evident through improvement recorded using outcome measures over the course of two months. Hence, the aim of this program was achieved.

## References

[1] Iglesias-Rodríguez S, Domínguez-Prado DM, García-Reza A, Fernández-Fernández D, Pérez-Alfonso E, García-Piñeiro J, Castro-Menéndez M (2021). Epidemiology of proximal humerus fractures. J Orthop Surg Res.

[2] Bhadbhade V, Channabasava Channabasava (2019). Clinical profile of proximal humeral fractures. Natl J Clin Orthop.

[3] Salvini TF, Durigan JL, Peviani SM, Russo TL (2012). Effects of electrical stimulation and stretching on the adaptation of denervated skeletal muscle: implications for physical therapy. Rev Bras Fisioter.

[4] Ohanisian L, Brown N, White SD, Rubay D, Schwartz PM (2019). Persistent shoulder pain due to a suprascapular nerve injury in the setting of trauma. Cureus.

[5] Reece CL, Varacallo M, Dulebohn SC, Susmarski AJ (2024). Suprascapular Nerve Injury. http://www.ncbi.nlm.nih.gov/books/NBK559151/.

[6] Solomen S, Babu B, Muralidharan PC, Sreejith K, Gafoor SA (2021). Conservative management of brachial plexus injury through a structured rehabilitation protocol: a case report. RGUHS J Physiother.

[7] de Santana Chagas AC, Wanderley D, de Oliveira Ferro JK, Alves de Moraes A, Morais de Souza FH, da Silva Tenório A, Araújo de Oliveira D (2022). Physical therapeutic treatment for traumatic brachial plexus injury in adults: a scoping review. PM R.

[8] Chagas AC, Wanderley D, Barboza PJ, Martins JV, de Moraes AA, de Souza FH, de Oliveira DA (2021). Proprioceptive neuromuscular facilitation compared to conventional physiotherapy for adults with traumatic upper brachial plexus injury: a protocol for a randomized clinical trial. Physiother Res Int.

[9] Limthongthang R, Muennoi P, Phoojaroenchanachai R, Vathana T, Wongtrakul S, Songcharoen P (2014). Effectiveness and safety of home-based muscle electrical stimulator in brachial plexus injury patients. J Med Assoc Thai.

